# Case Report: Two cases of secondary malignant neoplasms following treatment in pediatric patients

**DOI:** 10.3389/fped.2026.1921934

**Published:** 2026-09-09

**Authors:** Yue Wang, Huan Zhang, Qiuling Miao, Li Li, Fang Xu, Xiaoning Mao, Xianping Jiang

**Affiliations:** 1 Department of Pathology, Shenzhen Children’s Hospital, Shenzhen, China; 2 Department of Laboratory Medicine, Shenzhen Children’s Hospital, Shenzhen, China

**Keywords:** ALCL, CCSK, chemotherapy, Ewing sarcoma (ES), pediatric patients, secondary malignant neoplasm, T-ALL

## Abstract

Among the various long-term iatrogenic sequelae associated with the treatment of pediatric and adolescent patients with malignancies, secondary malignant neoplasms (SMNs) are among the greatest concerns. Chemotherapeutic agents may increase the risk of SMNs. We present the first case of a patient initially diagnosed with T-cell acute lymphoblastic leukemia (T-ALL), who received 30 months of chemotherapy, including the alkylating agent cyclophosphamide, the topoisomerase II inhibitor daunorubicin, and subsequently developed cervical anaplastic large cell lymphoma (ALCL) three years after the initial diagnosis. We also present the second case of a patient initially diagnosed with clear cell sarcoma of the kidney (CCSK), who underwent 6 months of chemotherapy, including the alkylating agent cyclophosphamide, the topoisomerase II inhibitors etoposide and pirarubicin, together with 3 months of radiotherapy, and subsequently developed Ewing sarcoma of the lower leg five years after the initial diagnosis. The cases reported in this study expand the spectrum of reported SMNs following treatment and provide pathologists with additional references for establishing pathological diagnoses on the basis of patients’ medical histories and prior treatment regimens.

## Introduction

Among the various long-term iatrogenic sequelae associated with the treatment of pediatric and adolescent patients with malignancies, secondary malignant neoplasms (SMNs) are among the greatest concerns. Chemotherapeutic agents, such as alkylating agents and topoisomerase II inhibitors may increase the risk of SMNs (1). The two cases of post-chemotherapy SMNs reported in this study are rare, and no similar cases have been documented in the literature.

## Case presentation

We analyzed two cases of secondary malignant neoplasms following treatment in pediatric patients (Table 1).

**Table 1:** Characteristics of pediatric patients with secondary malignant neoplasms following treatment.

Patient	Age@ Dx(yrs)	Gender	PMN (site)	CT regimen	RT	SMN (site)	SMN in irradiated area	Months since PMN Dx	Months since PMN end-treatment
1	7.2	Male	T-ALL	VDLD CAT CAT + T, HD-MTX MDA	NO	ALCL (neck)	NO	35	5
2	7	Male	CCSK (right kidney)	VDC CE	YES	Ewing sarcoma (right tibia)	NO	59	50

Dx, diagnosis; PMN, primary malignant neoplasm; CT, chemotherapy; RT, radiotherapy; SMN, secondary malignant neoplasm; T-ALL, T-cell acute lymphoblastic leukemia; VDLD, vincristine, daunomycin, pegaspargase, and dexamethasone; CAT, cyclophosphamide, cytosine arabinoside, and mercaptopurine; CAT+, cyclophosphamide, cytosine arabinoside, mercaptopurine, vincristine, and pegaspargase; T, mercaptopurine; HD-MTX, high-dose methotrexate; MDA, methotrexate, dexamethasone, and cytosine arabinoside; ALCL, anaplastic large cell lymphoma; CCSK, clear cell sarcoma of the kidney; VDC, vincristine, pirarubicin, and cyclophosphamide; CE, carboplatin and etoposide; ES, Ewing sarcoma.

### Patient 1

A 7.2-year-old boy presented in December 2022 with a 2-day history of painful submandibular lymphadenopathy. A complete blood count showed a white blood cell count of 361 × 10^9^/L, a platelet count of 34 × 10^9^/L, and a hemoglobin concentration of 89 g/L. A peripheral blood smear indicated that immature lymphocytes accounted for 91% of peripheral blood cells. A bone marrow aspirate smear showed proliferative, relatively uniform lymphoblastic cells with round or irregular nuclei, coarse chromatin, inconspicuous nucleoli, and scant to moderately basophilic cytoplasm. POX cytochemical staining was negative in the lymphoblastic cells. Flow cytometric analysis demonstrated that abnormal cells accounted for 92.9% of nucleated cells, with positive expression of HLA-DR, CD45, TdT, CD2, sCD3, cCD3, CD7, and CD38, partial expression of CD34, CD5, CD33, and CD58, and negative expression of CD117, CD15, CD19, CD20, CD4, CD8, and MPO. Next-generation sequencing identified a *TCF7-SPI1* gene fusion and *NOTCH1* gene mutation, which were clearly associated with T-cell acute lymphoblastic leukemia (T-ALL), and mutations in *CCND3* and *SETD1B*, which might also be associated with T-ALL. These features were diagnostic of T-ALL (intermediate-risk).

Chemotherapy was initiated immediately after the definitive diagnosis was established in January 2023. The patient was treated according to the Chinese Children's Cancer Group study ALL 2020 (CCCG-ALL-2020) intermediate/high-risk regimen. During the induction phase, the patient received VDLD therapy (vincristine, daunorubicin, pegaspargase, and dexamethasone), CAT therapy (cyclophosphamide, cytosine arabinoside, and mercaptopurine), and CAT + therapy (cyclophosphamide, cytosine arabinoside, mercaptopurine, vincristine, and pegaspargase). Complete remission was defined by a proportion of immature lymphocytes of 0.5% in the bone marrow. During the consolidation phase, the patient received four cycles of mercaptopurine and high-dose methotrexate. Maintenance therapy started in September 2023. During chemotherapy, repeated lumbar punctures were performed combined with intrathecal injections of the triple-therapy regimen (methotrexate, dexamethasone, and cytosine arabinoside) to prevent central nervous system leukemia. Chemotherapy was completed in June 2025. During chemotherapy for T-ALL, the cumulative dose of daunorubicin administered to Patient 1 was 150.4 mg, and the cumulative dose of cyclophosphamide was 5.805 g.

**Figure 1:**
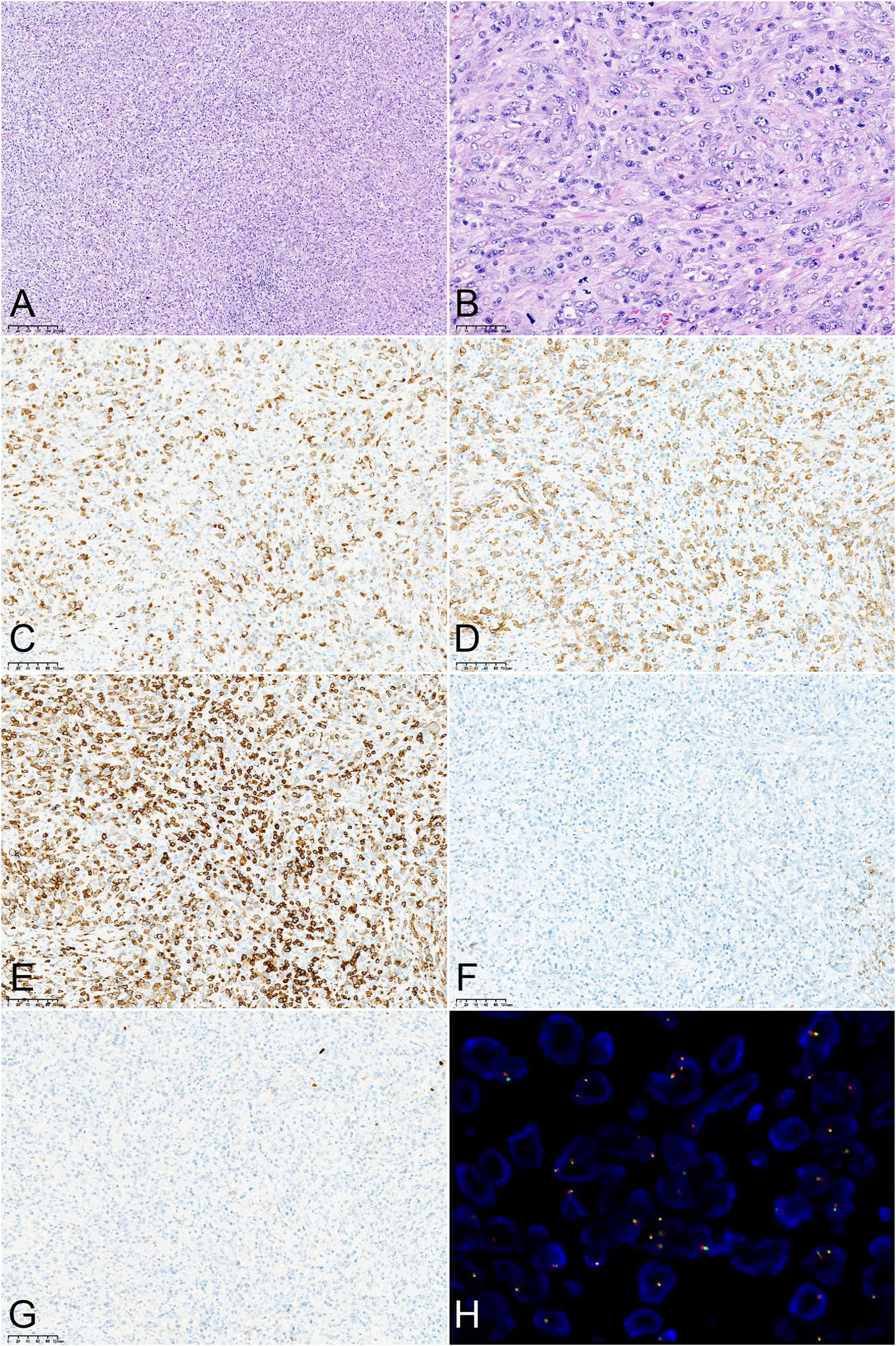
SMN in Patient 1. Histopathological and immunohistochemical features, and FISH examination of ALCL. **(A)** Low-power view showing diffuse infiltration of atypical neoplastic cells disrupting the normal lymph node architecture (H&E, 10×). **(B)** High-power view showing medium-to-large pleomorphic and anaplastic cells, some exhibiting hallmark cells with eccentric kidney-shaped nuclei (H&E, 40×). **(C–G)** IHC demonstrating cytoplasmic and membranous positivity for ALK **(C)**, membranous and cytoplasmic perinuclear Golgi-like staining for CD30 **(D)**, strong positivity for CD2 **(E)**, and negativity for CD20 **(F)** and TdT **(G)** in neoplastic cells (IHC, 20×). **(H)** Break-apart *ALK* fluorescence *in situ* hybridization showing translocation.

Five months after the completion of chemotherapy, in November 2025, the patient developed cervical lymphadenopathy. The lymph node sections showed replacement of the normal architecture by diffusely distributed atypical lymphoid cells, with round or oval nuclei showing vesicular morphology and visible nucleoli (Figure 1A). Mitotic figures were readily observed. Some nuclei displayed folding or lobulation, and some kidney-shaped nuclei highlighted hallmark cells (Figure 1B). The cytoplasm was abundant and appeared pale to faintly eosinophilic. Focal cells appeared spindle-shaped with fibrous septations. Small lymphocytic foci were present among the neoplastic cells. Immunohistochemical (IHC) staining revealed that neoplastic cells were positive for ALK (Figure 1C), CD30 (Figure 1D), CD2 (Figure 1E), and EMA; partially positive for CD3, CD4, CD7, CD8, CD43, CD99, and lysozyme; and negative for CD20 (Figure 1F), with TdT (Figure 1G) positivity limited to scattered lymphocytes. Break-apart *ALK* fluorescence *in situ* hybridization (FISH) revealed translocation (Figure 1H). Next-generation sequencing identified an *EEF1G-ALK* fusion. These features were diagnostic of ALK-positive anaplastic large cell lymphoma (ALCL).

Following definitive diagnosis, the patient has been receiving ALCL chemotherapy and has experienced chemotherapy-related diarrhea; no tumor recurrence has been observed.

### Patient 2

**Figure 2:**
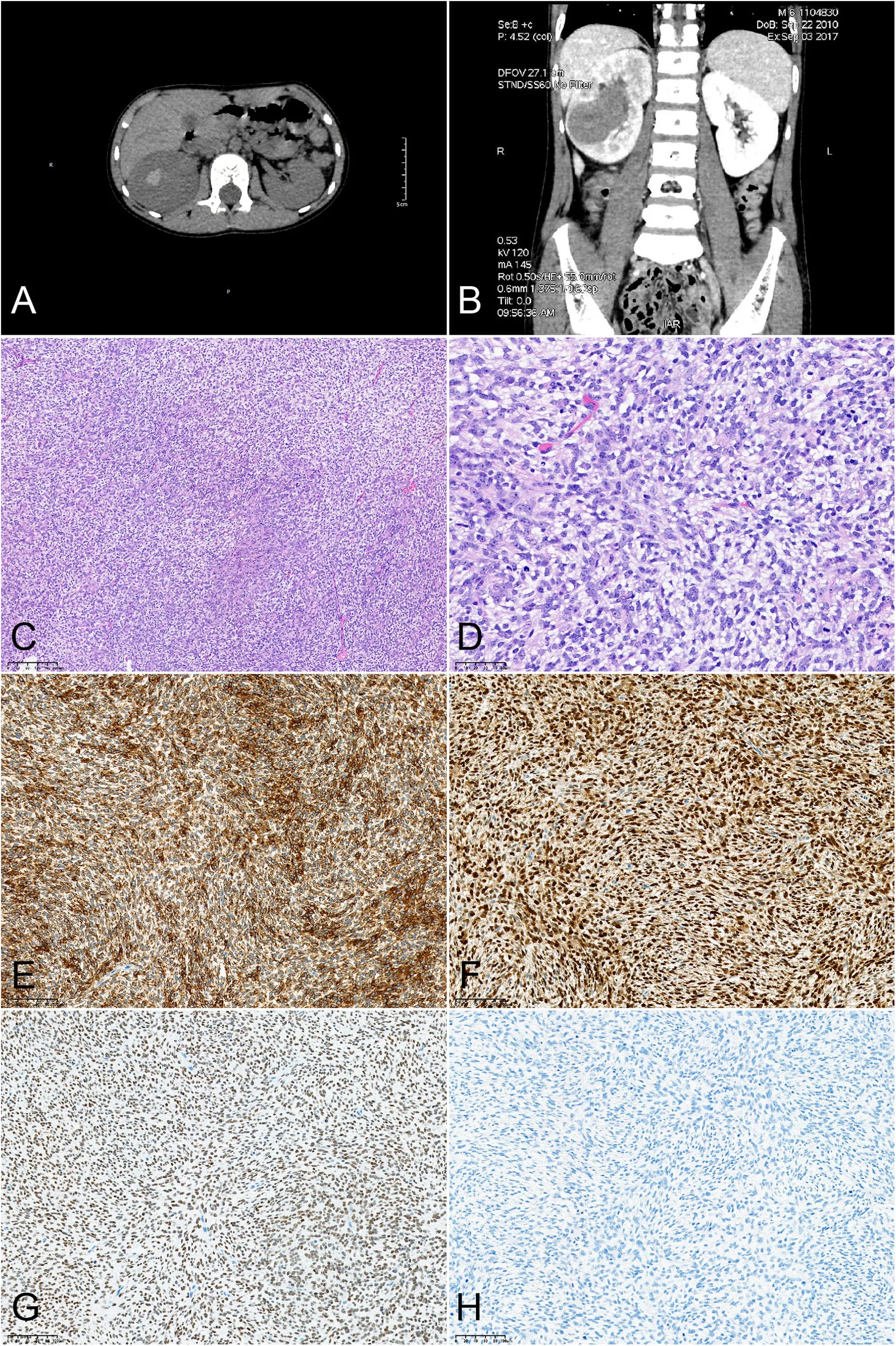
PMN in Patient 2. Radiological, histopathological, and immunohistochemical features of CCSK. **(A,B)** CT scan showing a large, circumscribed tumor mass in the renal medulla of the right kidney. **(C)** Low-power view showing that the tumor was primarily composed of spindle-shaped cells arranged in a sheet-like pattern and separated by arborizing fibrovascular septa, with some tumor cells exhibiting epithelioid characteristics and rosette-like structures (H&E, 10×). **(D)** High-power view showing round to oval tumor nuclei with small nucleoli, readily observable mitotic figures, and clear or faintly eosinophilic cytoplasm (H&E, 40×). **(E–H)** IHC demonstrating diffuse and strong positivity for Bcl-2 **(E)**, cyclin D1 **(F)**, and BCOR **(G)** in tumor cells, with negativity for WT-1 **(H)** (IHC, 20×).

A 7-year-old boy was admitted to the hospital in September 2017 due to hematuria for one day. Physical examination revealed percussion pain in the right renal region. A computed tomography (CT) scan showed a large, circumscribed tumor mass in the renal medulla of the right kidney (Figure 2A, B). The lesion measured approximately 34.1 mm × 44.7 mm × 52.0 mm (anteroposterior diameter×transverse diameter×longitudinal diameter) and had not penetrated the right renal capsule. Tumor mass excision was performed and showed that the tumor was primarily composed of spindle-shaped cells arranged in a sheet-like pattern and separated by arborizing fibrovascular septa (Figure 2C). Some tumor cells exhibited epithelioid characteristics and rosette-like structures. These round to oval tumor nuclei had small nucleoli and clear or faintly eosinophilic cytoplasm (Figure 2D). Mitotic figures were readily observed. IHC demonstrated diffuse and strong positivity for Bcl-2 (Figure 2E), cyclin D1 (Figure 2F), and BCOR (Figure 2G) in tumor cells, whereas WT-1 was negative (Figure 2H). On the basis of the clinicopathologic correlation, the tumor was interpreted as clear cell sarcoma of the kidney, staging I.

Chemotherapy was initiated immediately after the definitive diagnosis was established. The patient received vincristine, pirarubicin, and cyclophosphamide (VDC) in alternation with carboplatin and etoposide (CE), for a total of five cycles of VDC and four cycles of CE. The cumulative doses of chemotherapy administered to the patient were as follows: cyclophosphamide (4.47 g), pirarubicin (211 mg), and etoposide (343 mg). The patient achieved complete remission after completing chemotherapy in March 2018. From March to June 2018, the patient underwent radiation therapy in another hospital.

**Figure 3:**
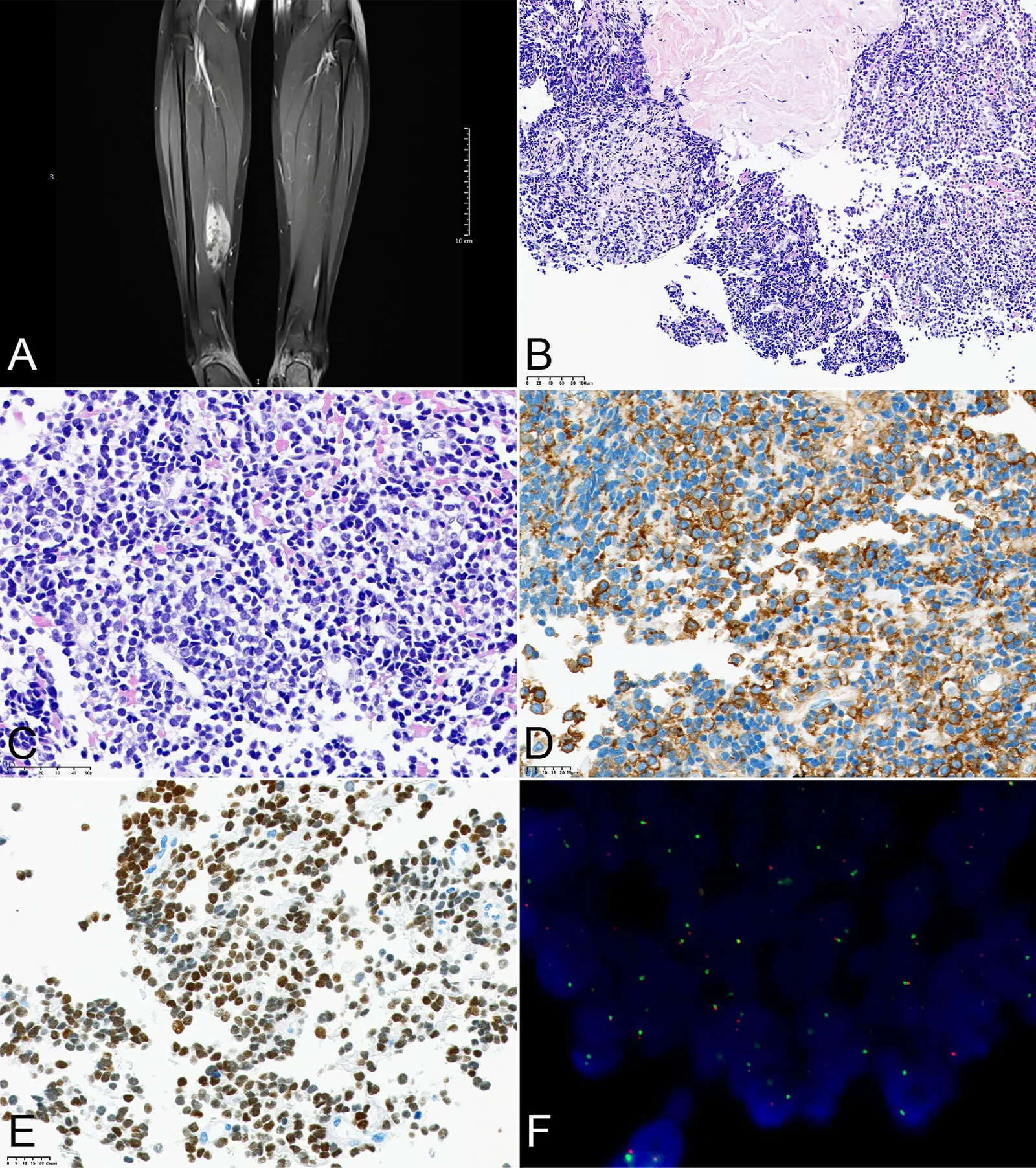
SMN in Patient 2. Radiological, histopathological and immunohistochemical features, and FISH examination of Ewing sarcoma. **(A)** MRI showing a tumor mass in the right tibia. **(B)** Low-power view showing a tumor composed of uniform small round cells infiltrating the surrounding soft tissue (H&E, 20×). **(C)** High-power view showing round nuclei with fine chromatin and mostly inconspicuous nucleoli, and tumor cells harboring scant clear or eosinophilic cytoplasm (H&E, 60×). **(D,E)** Consecutive sections demonstrating concurrent strong membranous positivity for CD99 **(D)** and nuclear positivity for NKX2.2 **(E)** (IHC, 60×). **(F)** FISH of the *EWSR1* gene revealing separate red/green signals, confirming *EWSR1* rearrangement.

Approximately four years after the completion of treatment, in August 2022, magnetic resonance imaging (MRI) revealed a patchy mass measuring 74 mm × 27 mm × 13 mm within the medial and inferior segment of the right tibia, with an ill-defined border; adjacent cortical bone showed uneven thickening and focal cortical destruction; no periosteal hyperplasia was observed; and a soft tissue mass with irregular margins was identified around the injury (Figure 3A). Biopsy of the tumor mass showed that the tumor was composed of uniform small round cells infiltrating the surrounding soft tissue (Figure 3B). The nuclei were uniform and round, with fine chromatin and mostly inconspicuous nucleoli (Figure 3C). The cytoplasm was clear to lightly eosinophilic and PAS-positive in special staining. The tumor cells did not form rosettes. IHC analysis revealed positive results for CD99 (Figure 3D), NKX2.2 (Figure 3E), Bcl-2, and cyclin D1, but negative result for BCOR. FISH examination of the *EWSR1* gene showed separate red/green signals, confirming *EWSR1* rearrangement (Figure 3F). Genetic testing revealed a positive *EWSR1-FLI1* fusion gene status. No cancer predisposition syndrome was identified. After clinicopathologic correlation, the tumor was interpreted as Ewing sarcoma, staging IIB.

Following a definitive diagnosis, the patient received preoperative chemotherapy for Ewing sarcoma, surgical resection of the tumor, and postoperative chemotherapy. After completion of treatment, pathological fractures and chronic osteomyelitis occurred. The patient's current condition is favorable, with no tumor recurrence or new malignant lesions observed.

## Discussion

SMNs are a major cause of late mortality in pediatric cancer survivors (2). The risk of SMNs in this population is 3–10 times higher than that in the general population, with risk factors including host genetics, primary cancer type, and exposure to chemotherapy or radiotherapy (3–6). A retrospective multicenter study conducted in Taiwan by Ho Wan-Ling et al. reported that among patients who developed SMNs in the cohort, acute myeloid leukemia (AML) was the most common SMN, followed by acute lymphoblastic leukemia (ALL), central nervous system tumors, and sarcomas (7). Koh K-N et al. retrospectively investigated 102 patients from 11 institutions who developed SMNs after pediatric cancer treatment between 1998 and 2011. The most common PMNs were central nervous system tumors, followed by ALL, non-Hodgkin lymphoma, and osteosarcoma. The most common SMNs were therapy-related myeloid neoplasms (AML and myelodysplastic syndromes), followed by thyroid carcinomas and central nervous system tumors (8). However, secondary lymphomas and sarcomas are less frequent, and their occurrence after specific primary cancers remains poorly characterized.

Children carrying specific germline genetic mutations have an elevated risk of developing myelodysplastic syndromes (MDS) or other hematopoietic malignancies (such as leukemia and lymphoma). Recent studies have identified an increasing number of such predisposition genes; mutations in these genes are most frequently observed in children with MDS, but they have also been detected in children diagnosed with other hematopoietic system malignancies. For some hematopoietic malignancy predispositions, particularly in children at high risk of developing MDS, early intervention via hematopoietic stem cell transplantation can have a positive impact on overall survival; this finding provides a theoretical basis for implementing rigorous clinical monitoring (9). An article indicates that CCSK does not appear to be associated with genetic predisposition syndromes and has not been reported to occur in individuals with germline genetic mutations. Likewise, familial cases of CCSK have not been reported (10). Based on the genetic testing results, neither of the two patients reported in this study has a cancer predisposition syndrome.

Patient 1 developed ALK-positive ALCL three years after completing chemotherapy for T-ALL, without prior radiotherapy. His treatment included the alkylating agent cyclophosphamide and the topoisomerase II inhibitor daunorubicin. Both drug classes are known to increase the risk of therapy-related myeloid neoplasms, typically presenting as acute myeloid leukemia (AML) or myelodysplastic syndrome (11–13). Therapy-related AML is associated with exposure to alkylating agents, which can induce partial or complete deletions of chromosome 5 or chromosome 7. Patients with topoisomerase II inhibitor-associated secondary AML often present chromosomal abnormalities involving chromosome band 11q23, such as *KMT2A* gene rearrangement (13). A series of studies on hematologic tumors following adjuvant chemotherapy for breast cancer have demonstrated a strong correlation between the use of chemotherapeutic agents and secondary hematologic malignancies (14–16). An article indicates that a higher risk of leukemia was restricted to patients who received a cumulative dose of cyclophosphamide more than 11,250 mg/m^2^ (17). However, secondary ALCL following T-ALL is extremely rare. To the best of our knowledge, this is the first report of ALK-positive ALCL as a second malignancy after T-ALL treated with chemotherapy alone. While secondary Hodgkin lymphoma has been reported after pediatric ALL (11, 12), the development of a CD30+ ALK + T-cell lymphoma in this setting expands the spectrum of chemotherapy-induced SMNs. The two hematological neoplasms occurring in Patient 1 differ significantly in both morphology and genetics. For patient with T-ALL, it is not always anaplastic lymphoma with bone marrow infiltration, and the most recent presentation is a relapse. The short latency (5 months after treatment completion) and absence of radiotherapy suggest a direct role of DNA-damaging agents in lymphoid transformation, possibly through off-target effects on progenitor cells.

Patient 2 developed Ewing sarcoma of the lower leg after treatment for CCSK, with the SMN located outside the radiation field. Radiotherapy was administered to an unspecified area, but the secondary tumor arose in the right tibia, which was not within the typical abdominal radiation field for renal tumors. According to Cahan's criteria, a post-irradiation sarcoma must lie within the irradiated area and have a latency >1 year (18). Thus, radiation is unlikely to be the primary cause in this case. Instead, the patient's chemotherapy regimen included cyclophosphamide (alkylating agent), etoposide, and pirarubicin (topoisomerase II inhibitors). These agents are known to increase the risk of secondary sarcomas, even in non-irradiated sites. Several case reports have described Ewing sarcoma (or primitive neuroectodermal tumor) after treatment for childhood ALL (19, 20), but secondary Ewing sarcoma after CCSK has not been previously reported. This case highlights that chemotherapy alone, without local radiotherapy, can induce a completely different sarcoma type in a distant site.

## Conclusion

In summary, these two rare cases broaden the phenotypic spectrum of SMNs after pediatric cancer treatment. Patient 1 illustrates that ALK-positive ALCL can occur as a pure chemotherapy-induced secondary malignancy, without prior radiotherapy. Patient 2 demonstrates that Ewing sarcoma may arise after CCSK treatment, more likely attributable to alkylating agents and topoisomerase II inhibitors. For pathologists, when evaluating a new tumor in a child with a history of cancer, a broad differential diagnosis including rare SMNs should be considered, even outside the radiation field. For clinicians, these cases underscore the importance of long-term surveillance for second cancers, not only hematologic but also solid, and not only within but also outside irradiated areas. Accurate staging assessment, together with comprehensive histopathological and molecular studies should be conducted to determine the appropriate chemotherapy regimen and avoid increasing toxicities in the short, medium, and long term.

## Data Availability

The original contributions presented in the study are included in the article/Supplementary Material, further inquiries can be directed to the corresponding author.
